# Number of maneuvers need to get a negative Dix-Hallpike test

**DOI:** 10.1016/S1808-8694(15)30512-7

**Published:** 2015-10-18

**Authors:** Nathali Singaretti Moreno, Ana Paula do Rego André

**Affiliations:** 1 Student of Speech and Hearing Therapist; 2 PhD. Speech and Hearing Therapy

**Keywords:** rehabilitation, dizziness, vertigo

## Abstract

Benign Paroxysmal Positional Vertigo is one of the most common causes of dizziness. Its characteristic clinical profile is dizziness at head movements. The main diagnostic maneuver of posterior canal Benign Paroxysmal Positional Vertigo is the Dix-Hallpike test. If the maneuver is positive (vertigo and/or nystagmus), the physician can perform the Epley maneuver on the injured side.

**Aim:**

This paper aims at checking the number of maneuvers necessary for patients with posterior canal Benign Paroxysmal Positional Vertigo to have a negative Dix-Hallpike test.

**Materials and methods:**

we carried out a retrospective analysis of 71 charts of patients with posterior canal Benign Paroxysmal Positional Vertigo, who were treated with the modified Epley maneuver.

**Study Design:**

Cross-Sectional Retrospective.

**Results:**

We found that 76.00% of the patients analyzed had the symptoms completely resolved and negative Dix-Hallpike test with a single maneuver.

**Conclusion:**

Based on our results it is possible to conclude that the number of modified Epley maneuvers is variable depending on the etiology, being that the Benign Paroxysmal Positional Vertigo secondary to the traumatic brain injury needed a greater number of maneuvers for Dix-Hallpike test to become negative.

## INTRODUCTION

The Benign Paroxysmal Postural Vertigo (BPPV) is one of the most common causes of dizziness^1^, ^2^, ^3^. Its characteristic clinical aspect is vertigo triggered by head movements, such as, for example, bending over, looking upwards, laying down or standing up from bed and roll in bed^4^, ^5^.

Pathophysiological theory of cupulolithiasis was described by Schuknecht. Such theory states that otolithic fragments detach from the utricle macula and stick to the semi-circular canal cupule, which stops working as angular acceleration transducer and starts working as linear acceleration transducer^6^. Now, the canaliculo lithiasis theory explains that the fragments do not remain adhered to the semi-circular canal cupula, but rather they float in the endolymph. Thus, the patient's head movement causes these fragments to move and thus an inadequate stimulation of the canal cupula, generating vertigo symptoms^7^.

This disease of the peripheral vestibular system affects mainly the posterior semicircular canal (PSC); however, it might also affect the other canals simultaneously^8^, ^9^. It may be associated to vestibular neuritis, Ménière's disease, and other diseases^10^; nonetheless, it seems idiopathic in most of the cases^5^. It can be found in all the age ranges and its prevalence increases with age^4^.

Positioning nystagmus study (characteristic eye movement) allows one to locate the damaged side and canal, and the distinction between canaliculo lithiasis and cupulolithiasis, being important to guide the best rehabilitation exercises for each case - a fundamental part of treatment. Vestibular rehabilitation exercises and maneuvers in BPPV depend on the injured canal and are specific for each one of them^11^.

The main BPPV diagnostic maneuver for the posterior canal is the Dix-Hallpike test, which aims at triggering the labyrinth symptom or signal such as vertigo, nausea and/or nystagmus. In cases of a positive maneuver (presence of vertigo and/or nystagmus), we indicate the Epley repositioning maneuver on the damaged side^12^ and the present work aims at checking the number of maneuvers necessary to have a negative Dix-Hallpike test, according to BPPV etiology.

## MATERIALS AND METHODS

The project was approved by the Institution's Ethics Committee under protocol # 5196/2007.

The present study was carried out by the chart analysis of 71 patients with otorhinolaryngological diagnosis of posterior canal Benign Paroxysmal Postural Vertigo (BPPV) treated with the modified Epley maneuver, carried out without the use of a bone vibrator on the mastoid, which were discharged from the Speech and Hearing Therapy Department of the State of São Paulo Public Hospital.

Exclusion criteria: Individuals diagnosed with posterior canal BPPV and those submitted to any other type of treatment which was not the modified Epley maneuver; horizontal or superior canal BPPV.

All the data collected was analyzed and plotted. For data statistical analysis we used the GENMOD procedure of the SAS 9.1 software.

A Poisson model was used in order to perform the counting of the number of maneuvers used for each treatment, comparing them to the age and gender of the patients involved in the study, as well as BPPV etiology. P values < 0.05 were considered significant.

## RESULTS

Of the 71 charts analyzed, 46 were females and 25 were males. Patient mean age was 54.87 years, and their age varied between 30 and 86 years. In terms of the laterality of affected canals, we found the involvement of 38 right side posterior canals and 33 left-side posterior canals.

In order to facilitate the comparison of age with the number of maneuvers used, the patients were grouped in three age categories: 30 to 50 years, 51 to 70 years and 71 to 90 years of age.

It is worth stressing that in this study we did not find patients who required three modified Epley's maneuvers, and we noticed the need for one, two or four maneuvers.

As far as BPPV etiology is concerned, we noticed the occurrence of head injury, hormonal changes, metabolic alterations and idiopathic cases.

In comparing the number of maneuvers and gender variables, we could observe that from the patients who were successful in posterior canal BPPV treatment with a single modified Epley Maneuver, 32 (59.26%) were females and 22 (40.74%) were males. Of the patients who remained symptomatic after the first modified Epley Maneuver and obtained positive results with the second maneuver, 13 (86.67%) were females and two (13.33%) were males. Finally, the patients who required four maneuvers, one (50.00%) was female and one (50.00%) was male (Table 1). In comparing genders, there was no statistically significant difference in relation to the number of maneuvers, and we noticed a p value equals; 0.0602.

**Table 1:** Crossing the number of maneuvers and gender variables

Number of Maneuvers						
Gender		1	2	3	4	Total
Females	n	32	13	0	1	46
	%	59,26	86,67	00,00	50,00	
Males	n	22	2	0	1	25
	%	40,74	13,33	00,00	50,00	
Total		54	15	0	2	71

In the number of maneuvers and age variables we could see that among the patients who were successful with the one maneuver treatment, 23 (42.59%) had between 30 and 50 years of age, 22 (40.74%) were between 51 and 70 years and nine (16.67%) were between 71 and 90 years. Of the patients who required two maneuvers, seven (46.67%) were between 30 and 50 years, five (33.33%) were between 51 and 70 years and three (20.00%) were between 71 and 90 years. In relation to the use of four maneuvers, one patient was between 30 and 50 years and the other was between 71 and 90 years. None of the patients between 51 and 70 years required four maneuvers (Table 2). Comparing the age ranges adopted, we did not observe statistically significant differences in relation to the number of maneuvers. Among individuals between 30 and 50 and 51 and 70 years we observed a p value equals; 0.2460; between 30 and 50 and 71 and 90 years, p equals; 0.1714; and, between 51 and 70 and 71 and 90 years, p equals; 0.7931.

**Table 2:** Crossing the number of maneuvers and age variables

Number of maneuvers						
Age (years)		1	2	3	4	Total
30-50	n	23	7	0	1	31
	%	42,59	46,67	00,00	50,00	
51-70	n	22	5	0	0	27
	%	40,74	33,33	00,00	50,00	
71-90	n	9	3	0	1	13
	%	16,67	20,00	00,00	50,00	
Total		54	15	0	2	71

In comparing the etiology and treatment variables we can see that the Epley maneuver was used in two (3.70%) of the patients with head injury, in eight (14.81%) of the patients with hormonal alteration, in 10 (18.52%) of the patients with metabolic alteration and in 34 (62.96%) of the patients considered idiopathic. They required two maneuvers: four (26.67%) of the patients with head injury, one (6.67%) patient with hormonal alteration, four (26.67%) patients with metabolic alteration and six (40.00%) patients with idiopathic cause. Of the patients who required four maneuvers, two (100.00%) had head injuries (Table 3).

**Table 3:** Crossing the number of maneuvers and etiology variables

Number of maneuvers						
Etiology		1	2	3	4	Total
Head Injury	n	2	4	0	2	8
	%	3,70	26,67	00,00	100,00	
Hormonal alteration	n	8	1	0	0	9
	%	14,81	6,67	00,00	00,00	
Metabolic alteration	n	10	4	0	0	14
	%	18,52	26,67	00,00	00,00	
Idiopathic	n	34	6	0	0	40
	%	62,96	40,00	00,00	00,00	
Total		54	15	0	2	71

We observed a statistically significant difference when we compared, within the etiology variable, head injury with hormonal alteration (p equals; 0.0015) and head injury with idiopathic cases (p < 0.0001). When head injury was compared with metabolic alteration we did not observe statistically significant differences (p equals; 0.0239) in relation to the number of maneuvers. We also did not observe any statistically significant difference when we compared hormonal alteration and metabolic alteration (p equals; 0.3334), hormonal alteration and idiopathic causes (p equals; 0.9347) and, still, metabolic alteration and idiopathic causes (p equals; 0.1781).

In general, 54 (76.00%) of the 71 patients analyzed had complete symptom resolution and negative Dix-Hallpike test with the use of one single modified Epley maneuver, 15 (21.00%) required two maneuvers and 2 (3.00%) required four maneuvers.

## DISCUSSION

BPPV is a highly prevalent labyrinth disorder which considerably impairs the patient's quality of life, making him/her social and/or professionally handicap^13^.

In the present study, most of the patients analyzed had a complete symptom resolution and Dix-Hallpike test resolution with only one modified Epley Maneuver. Our data are in accordance with the literature, which reference stress the success of such maneuver^14^, ^15^, ^16^, ^17^, ^18^, ^19^.

We found a predominance of this vestibular disease among females when compared to males, which is also in agreement with the literature studied - which reports the same prevalence^13^, ^14^, ^15^,^17^,^20^, ^21^, ^22^, ^23^.

The age variation found in our population shows that BPPV can affect youngsters, adults and the elderly, and it was similar to the variation found in other studies^14^,^15^,^19^,^24^.

BPPV was idiopathic in most of the patients in this study, which was also found in the literature^18^; however, it can also be secondary to hormonal or metabolic alterations or head injuries^4^.

All the individuals in our population were treated by the modified Epley maneuver. In the gender distribution analysis we did not observe any statistically significant difference, which indicates that both genders presented answers which were found in the literature, which also did not find differences between the genders in relation to the number of maneuvers utilized^23^.

In analyzing the individual age range and the number of maneuvers utilized, our study showed that, although the elderly were more predisposed to the disease, we observed that in all the age ranges studied the maneuver efficacy was the same. Some authors also observed the same efficacy concerning the repositioning maneuver in all the age ranges they studied. ^15^, ^23^

As far as etiology is concerned, we have observed that metabolic and hormonal alterations and idiopathic causes have between 70 and 90% of success with only one Eplay modified maneuver. However, when head injury was the cause, we observed that 25% of the patients required only one maneuver and the majority (50%) required two repositioning maneuvers, which is in agreement with some studies saying that head injury patients do nor respond favorably to the maneuver as it happens with the idiopathic causes^22^, ^25^. Other authors have also reported in their studies that idiopathic BPPV has a better response to treatment then when it is associated with other diseases^21^.

In this paper we used the Epley modified response without association to other treatments; however, the literature reports that the number of maneuvers necessary to extinguish the BPPV is variable and that more than one third of all the patients may require multiple treatments^26^.

## CONCLUSION

Having these results, we can then conclude that the number of modified Epley maneuvers varies according to etiology, and BPPV secondary to head injury required more maneuvers in order to turn negative the Dix-Hallpike test.
